# Metabolic Syndrome Prevalence and Its Components in Adolescents from Western Mexico

**DOI:** 10.3390/pediatric17040083

**Published:** 2025-08-08

**Authors:** Diego Ortega-Pacheco, Roberto Carlos Rosales-Gómez, Teresa Arcelia García-Cobián, Lidia Ariadna Rubio-Chávez, Angélica Adriana Gutiérrez-Rubio, José Hugo Rivera-Ramírez, Susan Andrea Gutiérrez-Rubio

**Affiliations:** 1 Instituto de Terapeutica Experimental y Clínica, Departamento de Fisiología, Centro Universitario de Ciencias de la Salud, Universidad de Guadalajara, Guadalajara C.P. 44340, Jalisco, Mexico; diego.opacheco@alumnos.udg.mx (D.O.-P.); arcelia.garcia@academicos.udg.mx (T.A.G.-C.); lidia.rubio@alumnos.udg.mx (L.A.R.-C.); 2 Laboratorio de Fisiología, Departamento de Ciencias Biomédicas, Centro Universitario de Tonalá, Universidad de Guadalajara, Tonala C.P. 45425, Jalisco, Mexico; roberto.rosales@academicos.udg.mx; 3 Departamento de Pediatría, Hospital de Ginecología y Obstetricia UMAE 25, Instituto Mexicano del Seguro Social, Monterrey C.P. 64180, Nuevo León, Mexico; bbloque9@gmail.com; 4 Departamento de Urgencias, Unidad Médica Familiar 68, Instituto Mexicano del Seguro Social, Guadalupe C.P. 67190, Nuevo León, Mexico; dr.hugo85@gmail.com

**Keywords:** adolescence, cardiometabolic risk, metabolic syndrome, primary prevention

## Abstract

Objective: To evaluate the predictive risk factors associated with the prevalence of metabolic syndrome (MetS) in adolescents from the western region of Mexico. Materials and Methods: An analytical cross-sectional study was conducted with a sample of 516 adolescents. Anthropometric data, blood pressure, and biochemical determinations were obtained. The diagnosis of MetS was based on the criteria proposed by de Ferranti in 2004. The triglyceride–glucose index (TyG index) was calculated, where a value >4.68 indicates insulin resistance (IR). Risk factors associated with MetS were evaluated using a logistic regression model. The statistical analysis was performed with the level of statistical significance established was *p* < 0.05. Results: The MetS prevalence was 17.2% among adolescents. One out of every two adolescents with obesity presented with MetS. Abdominal obesity and dyslipidemia are the most common components. Predictors of MetS included male sex, early adolescence, waist-to-height ratio (WHtR) > 0.5, increased body fat percentage, and TyG Index > 4.68 (IR). In the sex-specific analysis, a WHtR > 0.5 and IR were associated with MetS in female adolescents. In male adolescents, IR and body fat percentage were associated with MetS. The WHtR was associated with IR, and hypertriglyceridemia was associated with elevated alanine aminotransferase. Conclusions: In this study, two out of ten adolescents presented with MetS. In boys, a high prevalence of abdominal obesity, hypoalphalipoproteinemia, insulin resistance and MetS was observed. The risk of developing MetS is greater in preadolescent boys with abdominal obesity, high levels of body fat, and a TyG index > 4.68.

## 1. Introduction

Cardiovascular diseases (CVD) are the leading cause of mortality worldwide. In 2020, Latin America and the Caribbean reported a mortality rate of 218 deaths per 100,000 individuals attributed to CVD [1]. In Mexico, 200,535 deaths due to CVD were recorded in 2022, with ischemic heart disease accounting for 76.4% of cases, followed by hypertensive patients [2].

Obesity is the major risk factor for the development of CVD, and it continues to increase among adolescents [3]. Between 2006 and 2022, the prevalence of overweight and obesity in Mexican adolescents rose from 2.6% and 5.3%, respectively [4]. This trend is compounded by a high frequency of sedentary behaviors and increased consumption of high-caloric foods and sugary drinks within this age group. These factors contribute to an obesogenic environment that promotes increased body fat, particularly visceral adiposity [5,6].

Excess visceral adiposity is directly associated with chronic low-grade inflammation, insulin resistance (IR), dyslipidemia, high blood pressure (HBP), hyperglycemia, and metabolic syndrome (MetS) [7]. If these conditions are not diagnosed and managed early, they can progress to type 2 diabetes mellitus (T2DM) and CVD, significantly reducing quality of life and increasing health burdens. Therefore, identifying clinical characteristics indicative of cardiometabolic risk is a crucial preventive health strategy [8].

MetS is diagnosed when at least three of the following clinical features are present: elevated waist circumference, hypertriglyceridemia (HTG), decreased high-density lipoprotein cholesterol (HDL-C), HBP, and altered fasting blood glucose [9]. Given the ongoing obesity epidemic among children and adolescents, the increasing prevalence of MetS is unsurprising [10]. Early detection of MetS and its associated risk factors is essential to prevent comorbidities, reduce the economic burden associated with managing CVD and T2DM, and improve overall quality of life [11].

In this study, we aimed to evaluate the predictive risk factors associated with the prevalence of MetS in adolescents from the western region of Mexico.

## 2. Materials and Methods

### 2.1. Study Design and Participants

A cross-sectional analytical study was conducted on 516 adolescents from the general population, aged 10 to 17 years, after applying exclusion criteria that included any diagnosis of chronic illness, pregnancy, use of medications affecting metabolic parameters, or incomplete clinical or laboratory data. All participants, along with their legal guardians, signed an informed consent. General data, sociodemographic characteristics, history of addictions, level of physical activity, current diagnoses of cardiometabolic conditions, ongoing treatments, and family history of cardiometabolic diseases were recorded. Further details about the health campaign are available at https://sites.google.com/academicos.udg.mx/campaaportucorazn/inicio (accessed on 1 August 2025).

### 2.2. Sample Size

The sample size was calculated using Epi Info™ (Centers for Disease Control and Prevention, CDC, Atlanta, GA, USA; available at https://www.cdc.gov/epiinfo/index.html [accessed on 30 July 2025]), based on the prevalence of MetS reported in Mexican American adolescents: 13% in those with normal weight and 30% in those with overweight/obesity [12]. A statistical confidence level of 99%, a power of 80%, and a 5:1 control-to-case ratio were applied. A total sample size of 387 participants was initially calculated, with an estimated distribution of 65 adolescents with MetS and 322 without MetS. However, the final sample included 516 participants, of which 88 adolescents met the criteria for MetS and 428 did not. Given the phenotypic variability during adolescence, participants were stratified into three developmental stages: preadolescence (10–11 years), early adolescence (12–14 years), and late adolescence (15–17 years) [13]. To ensure representativeness across developmental stages, the sample size was proportionally estimated as one-third of the total participants per stage, resulting in a target of 129 adolescents for each stage. The final sample included 187 preadolescents, 164 early adolescents, and 164 late adolescents.

### 2.3. Anthropometric Data, Blood Pressure, and Biochemical Parameters

Weight and body fat percentage were measured using a Tanita BC-558 scale, while height was assessed with a SECA (SECA, Hamburg, Deutschland) 213 stadiometer. Waist and hip circumferences were measured using SECA (SECA, Hamburg, Deutschland) 201 measuring tapes. Body mass index (BMI), waist-to-height ratio (WHtR), and waist–hip ratio (WHR) were calculated.

Blood pressure (BP) was measured three times using an OMRON HEM-7120 (OMRON Corporation, Kyoto, Japan) sphygmomanometer. In cases of abnormal initial readings, follow-up measurements were scheduled one week later to confirm the results [14].

For biochemical determinations, a peripheral blood sample was collected in fasting of 10 to12 h, using a 5.0 mL BD vacutainer tube (catalog number 368159). Samples were centrifugated at 2500 rpm for 15 min to obtain serum, which was analyzed using ERBA (Erba Mannheim, London, UK) 180 equipment. Serum alanine aminotransferase (ALT), aspartate aminotransferase (AST), glucose, triglycerides, total cholesterol, and HDL-C were measured using Biosystems reagents, calibrators, and quality control serum.

Low-density lipoprotein (LDL-C) cholesterol was estimated using the Friedewald formula: LDL-C = Total cholesterol − (triglycerides/5) − HDL-C [15].

Very low-density lipoprotein (VLDL-C) cholesterol was calculated as follows: VLDL-C = Total cholesterol/5.

The TyG index was calculated using the following formula: ln(triglycerides (mg/dL) × glucose (mg/dL))/2. A value >4.68 was considered indicative of IR [16].

### 2.4. Diagnosis of Metabolic Syndrome and Its Components

MetS diagnosis was based on ATP III [17], Cruz [18], and de Ferranti [12] criteria. The de Ferranti criteria were selected for the analysis of the participants’ cardiometabolic factors. The de Ferranti criteria include the following cut-off points: waist circumference greater than the 75th percentile, systolic and diastolic blood pressure greater than the 90th percentile, fasting blood glucose greater than 110 mg/dL, serum triglycerides greater than 100 mg/dL, and serum HDL-C levels less than 50 mg/dL (for males aged 15–19 years, HDL-C value less than 45 mg/dL) [19].

### 2.5. Ethical Considerations

The study was approved by the Ethics Committee of the University Center for Health Sciences and conducted in accordance with the principles of the Declaration of Helsinki for research involving humans. The study’s registration number is CONBIOÉTICA-14-CEI-002-20191003.

### 2.6. Statistical Analysis

Qualitative data were expressed as frequencies and percentages, and comparisons were made using the chi-square test of independence. Quantitative data were presented as means and standard deviations (SD) and compared using either Student’s *t*-test or the Mann–Whitney U test, depending on the data distribution. A non-parametric correlation between quantitative data was performed. The logistic regression analysis (Wald backward stepwise method) was performed to identify predictor variables associated with MetS. All statistical analyses were conducted using IBM SPSS version 26, with a significance level of *p* < 0.01.

## 3. Results

### 3.1. Sociodemographic Characteristics, Lifestyle, and Preexisting Cardiometabolic Conditions

Many adolescents included in this study resided in urban areas, and only a small proportion reported alcohol and tobacco consumption. A high prevalence of sedentary lifestyles was observed, with female adolescents representing a significantly higher proportion (*p* < 0.004). Family history data indicated that 59.5% of participants had at least one relative with a cardiometabolic disease. Prior to this study, 9.5% of adolescents had been diagnosed with cardiovascular risk factors, including overweight/obesity, hypertension (HTN), T2DM, dyslipidemia, or polycystic ovary syndrome (PCOS). Detailed information is provided in Table 1.

### 3.2. Metabolic Syndrome, Its Components and Related Parameters

**Figure 1:**
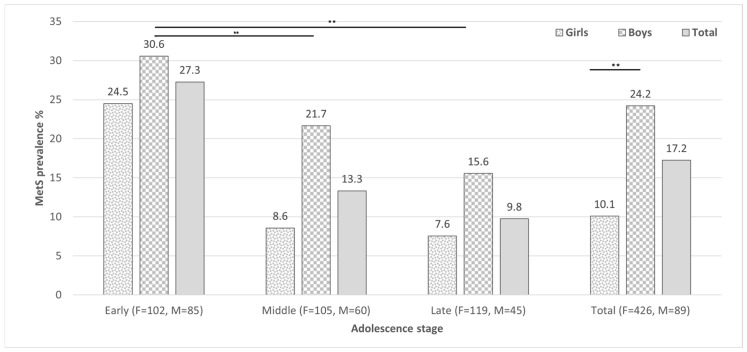
Metabolic syndrome prevalence stratified by sex for each stage of adolescence and in the overall population. ** *p* < 0.01.

The overall prevalence of MetS among participants was 17.2% by de Ferranti criteria (Figure 1), with a significantly higher proportion in male adolescents (*p* = 0.001). The most frequent MetS components were central obesity (CO), HTG, and low HDL-C levels. Male adolescents showed a higher frequency of abdominal obesity (*p* = 0.002), low HDL-C levels (*p* = 0.007), and HBP (*p* = 0.001). Notably, MetS was more prevalent during preadolescence compared to other stages of adolescence (*p* < 0.001).

**Figure 2:**
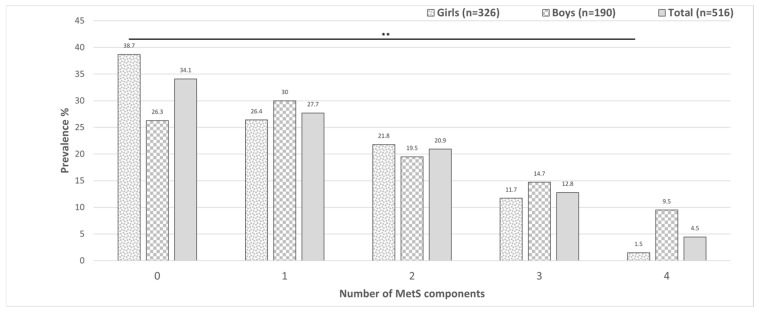
Prevalence of the number of metabolic syndrome components stratified by sex and among the overall 516 adolescents from Western Mexico. ** *p* < 0.01.

The proportion of adolescents without any MetS components was 34.1%, with female adolescents comprising most of this group (*p* = 0.004). Among participants, 27.7% had at least one MetS component, 20.9% had two components, 12.8% had three components, and 4.5% had four components. Male adolescents represented the largest proportion in all categories except the group with two components (Figure 2).

Adolescents with overweight and obesity (43.6%), determined as a BMI percentile >85th, had a significantly elevated risk of presenting with MetS (OR = 23.3; CI = 10.5–51.6; *p* < 0.001) compared to those with a BMI percentile <85th. When analyzed separately, obesity increased the risk of MetS (OR= 14.02; CI = 8.2–23.9; *p* < 0.001), while being overweight was not significantly associated with MetS (*p* > 0.05). Male adolescents exhibited the highest proportion of obesity (*p* = 0.007).

The frequency of IR surrogated was 14%, with a significantly higher prevalence in male adolescents (*p* = 0.049). All participants with IR had at least one MetS component. WHtR > 0.5 was associated with IR (OR = 2.71; CI = 1.22–6.02; *p* = 0.014). Furthermore, IR was positively correlated with serum levels of ALT (R = 0.290; *p* = 0.001), VLDL-C (R = 0.951; *p* < 0.001), and total cholesterol (R = 0.503; *p* < 0.001). Notably, 8.1% of adolescents had elevated ALT levels (>39 U/L). Male sex and IR were significantly associated with increased ALT levels (OR = 2.55; CI = 1.23–5.29; *p* = 0.012; OR = 2.09; CI = 1.09–3.97; *p* = 0.02, respectively).

### 3.3. Comparison of Quantitative Parameters and Risk Factors Associated with Metabolic Syndrome

Quantitative variables, except for glycemia (*p* > 0.05) and HDL-C (decreased levels), were significantly elevated in adolescents with MetS (Table 2). The WHtR was positively correlated with trunk fat percentage, corporal fat percentage, and BMI percentage. Additionally, triglycerides were correlated with trunk fat percentage, WHtR, and waist-to-hip ratio (WHR). Furthermore, the IR surrogated was correlated with all the variables; notable were the BMI, waist circumference, and WhtR (Table 2).

A logistic regression model was applied to identify factors associated with the high prevalence of MetS. The key predictors included WHtR > 0.5, IR, male sex, early adolescence, and increased body fat percentage. The model explained 29% of the variance (R^2^ = 0.29; Table 3).

In the sex-specific analysis, IR surrogated and WHR were significantly associated with MetS in female adolescents (*p* < 0.001). Among male adolescents, IR and a body fat percentage greater than the 75th percentile were the primary risk factors for MetS (*p* < 0.001).

## 4. Discussion

This study selected the de Ferranti criteria since they include more parameters, accurate value cut-off points, and are inflexible. Afterwards, in the adolescents studied, there was a high prevalence of cardiometabolic risk factors (CMRF), including overweight/obesity (43.6%), MetS (17.2%), CO, dyslipidemia, HBP, IR, and elevated transaminases. This epidemiological profile aligns with the findings reported in ENSANUT 2022 and other studies conducted in Mexico and globally [1,2,3,4,5,10,20].

Prior to this study, only 9.5% of adolescents had been diagnosed with clinical CMRF, highlighting a substantial under-detection compared to our findings. This discrepancy underscores the importance of systematic screening for cardiometabolic parameters and MetS in adolescents to enable early intervention and promote lifestyle modifications [11,20,21].

Abdominal obesity and dyslipidemia were the most prevalent components of MetS, consistent with previous studies using various diagnostic criteria [22,23]. Obesity remains the primary risk factor for the development of HBP and IR [3,4,5,6]. While blood pressure is generally preserved during early adolescence due to limited exposure to risk factors, our study revealed a moderately high prevalence of HBP, aligning with prior reports for this age group [24]. The development of HBP in adolescents is linked to obesity, sedentary lifestyles, stress, and high sodium intake, which can also exacerbate dyslipidemia and atherosclerosis [25,26,27].

The altered fasting glucose level was infrequent, likely due to compensatory hyperinsulinemia, where excess glucose is metabolized and stored as triglycerides in visceral adipose tissue [28]. Elevated triglyceride synthesis from excessive sugar consumption contributes to abdominal obesity, which was observed predominantly in male adolescents. This pattern reflects android fat storage, a more metabolically active and reactive distribution type [29].

Visceral fat accumulation represents a significant cardiovascular risk factor due to its high density of white adipocytes, which act as metabolic and immune regulators through the secretion of pro-inflammatory and anti-inflammatory adipokines [30]. Lipotoxicity induces cellular stress, including mitochondrial dysfunction, reactive oxygen species (ROS) production, and activation of the unfolded protein response (UPR) pathway. These mechanisms trigger low-grade inflammation and systemic insulin resistance, ultimately contributing to MetS, CVD, and T2DM [30,31].

In this study, the key predictors of MetS included male sex, early adolescence, CO (WHtR > 0.5), increased body fat percentage, and IR. These variables are inexpensive and straightforward to measure, making them practical tools for identifying adolescents at risk of MetS [25,26,27,28,29]. The TyG index emerged as a reliable marker for detecting IR and MetS, as it only requires fasting glucose and triglyceride measurements —both cost-effective tests. Since direct measurements of visceral fat are less reliable in adolescents, WHtR > 0.5 and body fat percentage can serve as more robust indicators of CO and its association with lipotoxicity [28,29,32].

The inflammatory markers reported in this study, ALT and AST, were not directly associated with MetS. However, ALT remains a relevant prognostic marker of liver inflammation, with a global prevalence of 7.6% in adolescents, consistent with the 8.1% observed in our study [32]. Male sex and elevated triglycerides were significantly associated with serum ALT levels >39 U/L. Elevated ALT in apparently healthy adolescents (>40 U/L) has been linked to a higher risk of developing non-alcoholic fatty liver disease (NAFLD) in later stages [33]. Lipotoxicity in hepatocytes can result in chronic inflammation and fibrosis if left unaddressed [29,30,31,32].

The findings of this study suggest that adolescents aged 10 to 13 years are likely not meeting recommended physical activity levels and exhibit high consumption of sugary beverages, energy-dense processed foods, and insufficient intake of healthy food groups. This behavior reflects the current obesogenic environment, as reported in ENSANUT 2022 [4,5,6].

Promoting healthy lifestyles in children and adolescents remains a priority, with emphasis on school and family environments as key intervention points. Raising awareness about healthy eating habits and the benefits of physical activity is critical. For instance, in 2023, Mexico approved regulations prohibiting the sale of junk food in school cooperatives, a measure that could positively impact adolescent health outcomes [34].

Future studies should consider assessing adipokines such as resistin, adiponectin, and leptin as direct biomarkers of low-grade chronic inflammation associated with visceral adiposity and MetS.

As limitations of the study, serum insulin levels were not determined, and the surrogate cut-off point for IR was validated primarily in adults. Additionally, Tanner stages of pubertal development were not evaluated, which may influence metabolic parameters during adolescence.

## 5. Conclusions

In adolescents from Western Mexico, the prevalence of metabolic syndrome is approximately one in five, with half of the participants with obesity presenting MetS. Central obesity and dyslipidemia were identified as the most frequent components. The association analysis revealed that male sex, preadolescence, waist-to-height ratio >0.5, TyG index > 4.68 (indicative of insulin resistance), and increased body fat percentage are significant predictors of MetS.

Both the waist-to-height ratio and the TyG index emerge as accessible, reliable, and highly sensitive tools for the early identification of metabolic syndrome in adolescents, making them a valuable clinical marker for systematic screening in school and community settings. Considering the high prevalence of this condition and its strong association with future cardiovascular disease and type 2 diabetes, implementing primary prevention strategies targeted at this age group is both necessary and crucial.

## Figures and Tables

**Table 1:** Sociodemographic, lifestyle, and clinical characteristics in 516 adolescents from Western Mexico.

Characteristic (Cut Point)	Total (*n* = 516) % (*n*)	Girls (*n* = 326) % (*n*)	Boys (*n* = 190) % (*n*)	*p*
Adolescence stage				
Pre-adolescence (10–11 years)	36.2 (187)	31.3 (102)	44.7 (85)	0.002
Early adolescence (12–14 years)	32 (165)	32.2 (105)	31.6 (60)	0.8
Late adolescence (15–17 years)	31.8 (164)	36.5 (119)	23.7 (45)	0.03
Residence area				
Urban	93.8 (484)	94.5 (308)	92.6 (176)	0.7
Rural	6.2(32)	5.5 (18)	7.4 (14)	0.7
Lifestyle				
Alcohol consumption	5.2 (27)	6.4 (21)	3.2 (6)	0.1
Tobacco consumption	1.9 (10)	2.3 (9)	0.5(1)	0.1
Sedentarism	31.8 (164)	36.2 (118)	24.2 (46)	0.004
Pathological history				
CVD Family history	59.5 (307)	61.7 (201)	55.8 (106)	0.2
Previous diagnosis of T2DM, obesity, or CVD	9.5 (49)	11 (36)	6.8 (13)	0.1
Treatment of T2DM, HBP, dyslipidemia	0.1 (5)	0.9 (3)	1.1 (2)	1
Treatment of mood disorders	1.2 (6)	0.9 (3)	1.6 (3)	0.7
Clinic characteristics				
MetS (NCEP-ATPIII) [17]	9.3 (46)	4.2 (13)	13.3 (24)	0.001
MetS (Cruz et al., 2004) [18]	7.5 (37)	5.4 (17)	16 (29)	0.001
Mets (de Ferranti et al., 2004) [12]	17.8 (88)	13.7 (43)	24.9 (45)	0.002
High waist circumference (Per > 75)	38.8 (200)	33.7 (110)	47.4 (90)	0.002
Hypertriglyceridemia (>100 mg/dL)	29.7 (153)	27.6 (90)	33.2 (63)	0.18
Low HDL-C ^1^	22.5 (116)	18.7 (61)	28.9 (55)	0.007
High blood pressure (Per > 90)	7.9 (41)	4.9 (16)	13.2 (25)	0.001
Altered fasting blood glucose (>110 mg/dL)	0.8 (4)	0.6 (2)	1.1 (2)	0.6
Overweight + obesity (BMI Per > 85)	43.6 (225)	41.1 (134)	47.9 (91)	0.1
Overweight (BMI Per > 85–95)	17.6 (91)	19.6 (64)	14.2 (27)	0.4
Obesity (BMI Per > 95)	26 (134)	21.5 (70)	33.7 (64)	0.007
High corporal fat (Per > 85)	12.8 (66)	16 (52)	7.4 (14)	0.005
High trunk fat (Per > 85)	9.8 (36)	7.1 (23)	6.8 (13)	0.1
Surrogate insulin resistance (TyG index > 4.68)	14 (72)	11.7 (38)	17.9 (34)	0.049
Elevated LDL-C (>100 mg/dL)	10.5 (54)	10.7 (35)	10 (19)	0.7
Elevated VLDL-C (>30 mg/dL)	8.3 (43)	6.4 (21)	11.6 (22)	0.04
High total cholesterol (>165 mg/dL)	26 (134)	28.8 (94)	21.1 (40)	0.05
Altered AST ^2^	31.4 (162)	32.2 (105)	30 (57)	0.6
Altered ALT ^3^	8.1 (42)	5.8 (19)	12.1 (23)	0.01

Differences between sexes were evaluated using the chi-square test. Data are shown in percentages and frequencies. Per: percentile, CVD: cardiovascular disease, BMI: body mass index, HDL-C: high-density lipoprotein, LDL-C: low-density lipoprotein, VLDL-C: very-low-density lipoprotein, TyG index: triglycerides–glucose index, GOT: glutamic oxalacetic transaminase, GPT: glutamic–pyruvic transaminase. ^1^ Low HDL-C: overall < 50 mg/dL, boys 15 to 19 years <45 mg/dL. ^2^ Elevated AST: girls: 12–24 U/L, boys: 13–32 U/L. ^3^ Elevated ALT: overall: 1–13 years: 9–23 U/L, girls 13–19 years: 8–21 U/L, boys 13–19 years: 9–22 U/L.

**Table 2:** Correlation of cardiometabolic parameters in a sample of Mexican adolescents.

Clinical/Biochemical Characteristic	Age (Years)	Physical Exercise	Weight (kg)	BMI (kg/m^2^)	Corporal Fat (%)	Trunk Fat (%)	WC (cm)	HC (cm)	WHR	WHtR	SBP (mm/Hg)	DBP (mm/Hg)	HDL-C (mg/dL)	TG (mg/dL)	FBG (mg/dL)	TyG Index	**LDL-C** (**mg/dL)**	**VLDL-C** (**mg/dL)**	**Chol** (**mg/dL)**	**AST** (**U/L)**	**ALT** (**U/L)**
Age (years)	1																				
Physical exercise	0.18 **	1																			
Weight (kg)	0.29 **	−0.01	1																		
BMI (kg/m^2^)	0.15 **	−0.09	0.89 **	1																	
Corporal fat (%)	0.01	−0.211 *	0.57 **	0.75 **	1																
Trunk fat (%)	−0.01	−0.25 **	0.55 **	0.69 **	0.92 **	1															
Waist circumference (cm)	0.05	−0.13 *	0.87 **	0.86 **	0.62 **	0.60 **	1														
Hip circumference (cm)	0.29 **	−0.08	0.90 **	0.88 **	0.69 **	0.66 **	0.82 **	1													
Waist–hip ratio	−0.26 **	−0.11 *	0.39 **	0.40 **	0.24 **	0.25 **	0.72 **	0.19 **	1												
Height–waist ratio	−0.10 *	−0.19 **	0.70 **	0.85 **	0.69 **	0.65 **	0.93 **	0.72 **	0.73 **	1											
SBP (mm/Hg)	0.01	0.02	0.44 **	0.33 **	0.10 *	0.13 *	0.41 **	0.35 **	0.27 **	0.30 **	1										
DBP (mm/Hg)	−0.02	−0.11 *	0.24 **	0.22 **	0.24 **	0.26 **	0.270 *	0.24 **	0.17 **	0.24 **	0.54 **	1									
HDL-C (mg/dL)	0.34 **	0.22 **	−0.19 **	−0.19 **	−0.15 **	−0.19 **	−0.31 **	−0.18 **	−0.32 **	−0.31 **	−0.21 **	−0.16 **	1								
Triglycerides (mg/dL)	−0.04	−0.09	0.32 **	0.33 **	0.27 **	0.23 **	0.37 **	0.27 **	0.29 **	0.35 **	0.14 **	0.17 **	−0.222 *	1							
FBG (mg/dL)	−0.08	0.01	−0.01	−0.02	−0.06	−0.05	0.03	−0.06	0.13 **	0.04	0.08	0.08	0.04	0.15 **	1						
TyG index	−0.09 *	−0.12 *	0.29 **	0.32 **	0.28 **	0.25 **	0.36 **	0.25 **	0.31 **	0.37 **	0.16 **	0.18 **	−0.22 **	0.90 **	0.33 **	1					
LDL-C (mg/dL)	−0.17 **	−0.14 *	0.13 **	0.21 **	0.23 **	0.24 **	0.21 **	0.13 **	0.19 **	0.26 **	0.10 *	0.17 **	−0.22 **	0.19 **	0.09 *	0.29 **	1				
VLDL-C (mg/dL)	−0.05	−0.09	0.32 **	0.33 **	0.27 **	0.23 **	0.37 **	0.27 **	0.29 **	0.35 **	0.14 **	0.17 **	−0.22 **	1.0 **	0.15 **	0.90 **	0.19 **	1			
Total cholesterol (mg/dL)	0.05	−0.02	0.11 *	0.17 **	0.19 **	0.17 **	0.11 *	0.09 *	0.07	0.14 **	**0.01**	0.11 *	0.34 **	0.36 **	0.15 **	0.41 **	0.79 **	0.36 **	1		
AST (U/L)	0.01	0.03	0.14 **	0.10 *	−0.01	0.01	0.14 **	0.08	0.13 **	0.11 *	0.12 **	0.07	**0.08**	0.18 **	0.43 **	0.23 **	0.09 *	0.18 **	0.19 **	1	
ALT (U/L)	0.15 **	0.05	0.22 **	0.18 **	0.04	0.05	0.18 **	0.17 **	0.10 *	0.14 **	0.10 *	0.04	0.13 **	0.14 **	0.31 **	0.19 **	0.12 **	0.14 **	0.23 **	0.71 **	1

BMI: body mass index, WC: waist circumference, HC: hip circumference, WHR: waist–hip ratio, WHtR: waist–height ratio, SBP: systolic blood pressure, DBP: diastolic blood pressure, HDL-C: high-density lipoprotein, TG: triglycerides, FBG: fasting blood glucose, TyG index: triglyceride–glucose index, LDL-C: low-density lipoprotein, VLDL-C: very-low-density lipoprotein, Chol: cholesterol, AST: aspartate aminotransferase, ALT: alanine aminotransferase. * *p* < 0.05, ** *p* < 0.01.

**Table 3:** Predictors of MetS in a sample of adolescents from Western Mexico.

Indicators	OR (CI 99%)	*p*
Male	3.22 (1.26–8.21)	0.001
WHtR > 0.5	7.22 (2.33–22.33)	<0.001
Early adolescence stage	1.98 (1.22–3.24)	<0.001
Corporal fat percentage	1.09 (1.02–1.17)	0.001
Subrogated IR	4.54 (1.86–11.03)	<0.001

WHtR: waist–height ratio, IR: insulin resistance.

## Data Availability

The original contributions presented in this study are included in the article. Further inquiries can be directed to the corresponding author(s).

## Abbreviations

The following abbreviations are used in this manuscript:

MetS	Metabolic Syndrome
BMI	Body Index Mass
TyG index	Triglyceride–Glucose Index
IR	Insulin Resistance
WHtR	Waist-to-Height ratio
CVD	Cardiovascular diseases
HBP	High Blood Pressure
T2DM	Type 2 Diabetes Mellitus
HTG	Hypertriglyceridemia
Per	Percentile
HDL-C	High-density Lipoprotein
LDL-C	Low-density Lipoprotein
VLDL-C	Very-low-density Lipoprotein
GOT	Glutamic Oxalacetic Transaminase
GPT	Glutamic Pyruvic Transaminase
ALT	Alanine Aminotransferase
AST	Aspartate Aminotransferase
SD	Standard Deviation
HTN	Hypertension
PCOS	Polycystic Ovary Syndrome
CO	Central Obesity
CMRF	Cardiometabolic Risk Factors
ROS	Reactive Oxygen Species
UPR	Unfolded Protein Response
NAFLD	Non-alcoholic Fatty Liver Disease
